# Convolutional Neural Network-Based Pattern Recognition of Partial Discharge in High-Speed Electric-Multiple-Unit Cable Termination

**DOI:** 10.3390/s24082660

**Published:** 2024-04-22

**Authors:** Chuanming Sun, Guangning Wu, Guixiang Pan, Tingyu Zhang, Jiali Li, Shibo Jiao, Yong-Chao Liu, Kui Chen, Kai Liu, Dongli Xin, Guoqiang Gao

**Affiliations:** 1 CRRC Qingdao Sifang Co., Ltd., Qingdao 266000, China; sunchuanming@cqsf.com (C.S.); panguixiang@cqsf.com (G.P.); 2 School of Electrical Engineering, Southwest Jiaotong University, Chengdu 611756, China; gnwu@home.swjtu.edu.cn (G.W.); tingyu@my.swjtu.edu.cn (T.Z.); ljl@my.swjtu.edu.cn (J.L.); shibojiao@my.swjtu.edu.cn (S.J.); liukai@swjtu.edu.cn (K.L.); xindonglihero@my.swjtu.edu.cn (D.X.); xnjdggq@swjtu.edu.cn (G.G.); 3 Energy Department, UTBM, Université Bourgogne Franche-Comté, 90010 Belfort, France

**Keywords:** high-speed electric multiple units, cable terminal, partial discharge, pattern recognition, convolutional neural network

## Abstract

Partial discharge detection is considered a crucial technique for evaluating insulation performance and identifying defect types in cable terminals of high-speed electric multiple units (EMUs). In this study, terminal samples exhibiting four typical defects were prepared from high-speed EMUs. A cable discharge testing system, utilizing high-frequency current sensing, was developed to collect discharge signals, and datasets corresponding to these defects were established. This study proposes the use of the convolutional neural network (CNN) for the classification of discharge signals associated with specific defects, comparing this method with two existing neural network (NN)-based classification models that employ the back-propagation NN and the radial basis function NN, respectively. The comparative results demonstrate that the CNN-based model excels in accurately identifying signals from various defect types in the cable terminals of high-speed EMUs, surpassing the two existing NN-based classification models.

## 1. Introduction

The cable of the high-speed electric multiple unit (EMU) plays a critical role in the power supply system of high-speed trains, directly impacting the safety of train operations. The terminal, as a vulnerable component of the high-speed EMU cable, is particularly susceptible to partial discharge (PD), which poses a threat to the efficient functioning of high-speed trains [1,2,3,4]. Detecting PD is crucial for assessing the insulation condition of cable terminals. By identifying local discharges, the extent of insulation deterioration can be determined, allowing for timely maintenance or replacement measures [5,6,7]. Currently, technologies for the PD detection encompass a variety of methods, including the pulse current method, the high-frequency pulse current method, the ultra-high frequency detection method, the ultrasonic detection method, the optical measurement method, and the infrared imaging technology [8,9,10,11,12,13,14]. The advantages and disadvantages of these PD detection methods, along with their applicable scopes, are presented in Table 1.

Currently, the pulse current method, while being the earliest and most widely used PD detection method under the International Electrotechnical Commission standard, has some limitations [15]. Firstly, it primarily captures the lower frequency band of the PD signal, failing to acquire complete frequency information, particularly the high-frequency components. This omission may result in the neglect or misinterpretation of crucial discharge patterns. Secondly, the pulse current method has limited resistance to interference in practical applications, making it susceptible to electromagnetic interference. This vulnerability leads to a high bit error rate and diminished detection efficacy [16,17,18]. In contrast, the high-frequency pulse current detection system boasts advantages such as the ease of installation, a straightforward design, no impact on the operational state of the high-speed EMU cable terminal, broader frequency range coverage of PD signals, heightened test sensitivity, and robust anti-interference capabilities. It enables a quantitative analysis of the discharge magnitude of PD signals [19,20]. Consequently, this paper employs the high-frequency pulse current method as the PD testing approach for the cable terminals of high-speed EMUs.

The PD, as a crucial indicator for assessing the condition of power equipment, has been incorporated into the testing standards by the International Electrotechnical Commission [21]. However, the pattern recognition and fault diagnosis of the PD of the cable require further exploration. Numerous factors contribute to the PD, and the locations and mechanisms of cable defects vary.

As a key component connecting the electric equipment in the vehicle to the external power supply, the cable terminal of high-speed EMUs exhibits unique characteristics in terms of operating environment, structural design, and performance requirements. It differs significantly from other types of cable terminals, such as those in the power grid. High-speed EMU cable terminals operate in complex environments characterized by high-speed movement, vibration, and severe temperature and humidity fluctuations. Consequently, they are subjected to more severe mechanical and environmental stresses than those experienced by power grid cable terminals. The demanding operating conditions also necessitate significant differences in the structure and insulation materials of vehicle cable terminals. Generally, these terminals utilize a heat-shrink structure that offers improved sealing, weather resistance, and durability. They are made from EPDM rubber (EPR) materials, which provide resistance to oxidation, heat, and aging [22,23].

Based on the anatomy of high-speed EMU cable terminals with on-site faults and the maintenance experience of on-site staff, faults in these terminals are generally categorized as long-term, short-term, and unpredictable. Among these, short-term faults are the most prominent, with typical defects including insulation scratches, interlayer air gaps, metal particles, and uneven semi-conductive layers [24]. Clarifying the cause and action mechanism of each type of discharge holds significant importance for the routine maintenance of high-speed EMU cables and the early warning of potential accidents.

With rapid advancements in artificial intelligence (AI), its techniques are increasingly being adopted for robust control, accurate diagnosis, reliable prognosis, and effective signal analysis in electrical apparatus and systems [25,26,27,28,29,30,31,32]. Researchers globally have delved into the development of AI-powered pattern recognition methods for the PD of high-voltage cables. Common pattern recognition methods include the artificial neural network (NN), the support vector machine (SVM), the Bayes criterion, and the cluster analysis [33,34,35,36,37,38]. In the realm of PD pattern recognition, the back-propagation NN (BPNN) and the radial basis function NN (RBFNN) are widely used. In [39], the creation of cable models with five types of defects and the establishment of a dataset based on the phase-resolved PD (PRPD) spectrum were detailed, and the convolutional NN (CNN) was employed to achieve successful recognition of defect signals. In [40], PD signals from power cables with five types of insulation defects were collected, and a set of parameters characterizing discharge characteristics was established. The study found that the CNN-based model outperformed the BPNN-based and SVM-based models in signal identification. In [41], a time-domain waveform image database of four kinds of PD defects was constructed, and image processing technologies such as image enhancement and normalization were used to process these waveform images, and a DenseNet model was built to realize the recognition of the four kinds of defects, and the model has good robustness. In [42], the PD and corona signals were collected from cable terminals, and a CNN-based model was used for the signal recognition. However, this study bypassed the construction of a feature dataset, opting to directly feed discharge signals into the model.

In this paper, a CNN-based PD signal classification model is proposed for high-speed EMUs, which enables the identification of discharge signals from cable terminals exhibiting four typical defects. The main contributions of this paper are summarized as follows:

1. Characteristic parameters of terminal discharge signals in high-speed EMU cables are not extracted; instead, the four types of discharge signals are directly used as input to the model, achieving high accuracy.
2. The impact of different training datasets on the classification performance of the terminal discharge signal recognition model for high-speed EMUs is compared and analyzed. The proposed CNN-based model is demonstrated to flexibly meet the varying requirements for processing time and accuracy across different scenarios.
3. The proposed recognition model for terminal discharge signals in high-speed EMU cables is compared with two existing NN-based models, and it is verified that the CNN-based model exhibits superior recognition effectiveness.

## 2. Experimental Data Acquisition

### 2.1. PD Test Platform

**Figure 1:**
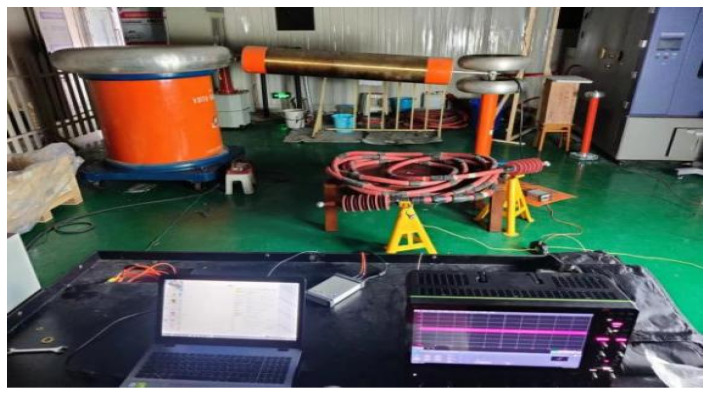
PD test platform for high-speed EMU cable terminals.

**Figure 2:**
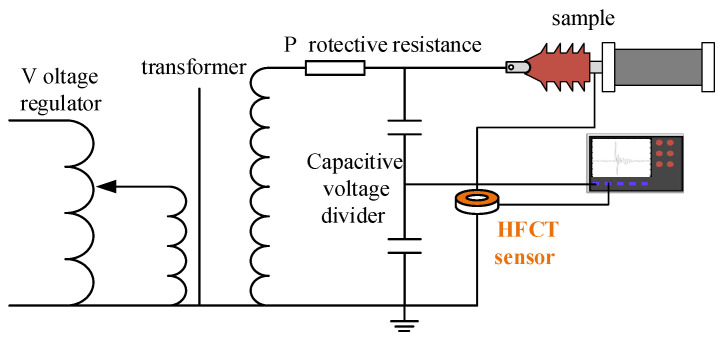
Circuit diagram of PD test platform based on the HFCT.

The PD test platform used in this study comprises the voltage regulator, the test transformer, the protection resistance, the discharge sample, the high-frequency current transformer (HFCT), and the high-frequency oscilloscope, among other components, as depicted in Figure 1. The HFCT, known for its exceptional sensitivity, straightforward setup, and strong anti-interference capabilities [43], is a widely used instrument for the online detection of PD signals, particularly when the ground conductor of the device under test is accessible. The circuit of the PD test platform is illustrated in Figure 2. By increasing the test voltage with the voltage regulator, insulation defect sample discharges are induced, thereby simulating PD defects [44].

### 2.2. Four Typical Defect PD Models

**Figure 3:**
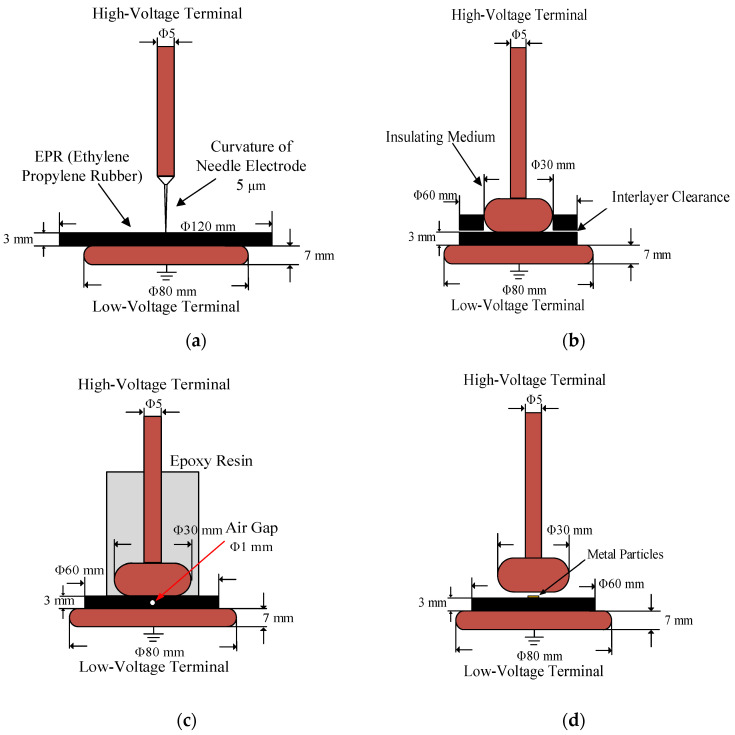
Electrode model of typical PD defect: (**a**) tip discharge, (**b**) surface discharge, (**c**) air gap discharge, and (**d**) suspension discharge.

During testing and operation, the cable terminal may exhibit discharge phenomena due to internal defects. The primary defect types include wire core burrs, surface sliding, internal air gaps, and suspended metal particles. To simulate these four discharge defects at the cable terminal, electrode structures for four typical discharge models are designed, as depicted in Figure 3. The tip discharge model simulates conductor burrs, which are difficult to eliminate completely during the fabrication of cable terminals and may cause discharge phenomena during operation. The surface discharge model simulates discharges caused by looseness or delamination between the insulation layers inside the cable terminal. The air gap discharge model simulates discharges caused by tiny bubbles or knife marks in the insulation layer during terminal operation. Lastly, the suspended discharge model simulates PD issues caused by conductive and semi-conductive impurities attached to the main insulation surface [45,46,47,48,49,50].

1. Tip discharge model: This model employs a steel needle with a curvature radius of 5 μm and uses ethylene–propylene–diene monomer (EPDM) rubber film as the insulating medium, with a diameter of 120 mm and a thickness of 3 mm. A ground electrode with a diameter of 80 mm is connected below the rubber film, and the steel needle is linked to a high-voltage electrode. The tip is inserted into the film to a depth of approximately 1 mm.
2. Surface discharge model: In this model, the insulating medium consists of an EPDM rubber film with a diameter of 60 mm, structured as a double layer, and has a total thickness of 6 mm. Below this, a ground electrode with a diameter of 80 mm is connected, and a copper disk with a diameter of 30 mm is positioned between the insulating medium and the high-voltage electrode.
3. Air gap discharge model: In this model, the insulating medium is again EPDM rubber film, with a diameter of 60 mm and a thickness of 3 mm. To simulate an air gap discharge, a circular hole with a diameter of 1 mm is created within the insulating medium. A copper disk is placed between the high-voltage electrode and the insulating medium. To avoid surface discharge interference, the high-voltage electrode in the air gap discharge model is sealed with epoxy resin.
4. Suspension discharge model: In this model, the insulating medium is EPDM rubber with a diameter of 120 mm and a thickness of 3 mm, and the high electric electrode is a copper disk with a diameter of 30 mm. There is a certain gap between the high electrode and the insulating medium, and a copper sheet with a thickness of 1 mm is placed in the gap as a suspended metal particle to simulate the suspended electrode.

### 2.3. High-Frequency Pulse Signals of PD with Four Typical Defects

**Figure 4:**
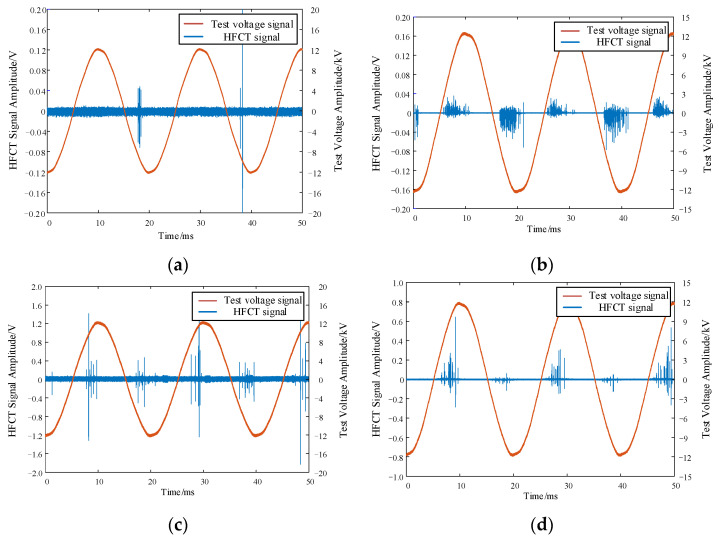
Time domain waveforms of discharge high-frequency pulse current signal of four defect models: (**a**) tip discharge, (**b**) surface discharge, (**c**) air gap discharge, and (**d**) suspended discharge.

The PD test platform shown in Figure 1 was used to carry out systematic pressure tests on four defect models mentioned above. The test protocol involved gradually increasing the voltage in 1 kV increments, maintaining each level for 1 min after pressurization to ensure test stability. High-frequency pulse current signals from the four defect models were collected within two power frequency cycles under a 12 kV test voltage. The time-domain waveforms of these signals are presented in Figure 4.

## 3. Design of Cable Discharge Classification Model

### 3.1. CNN

**Figure 5:**
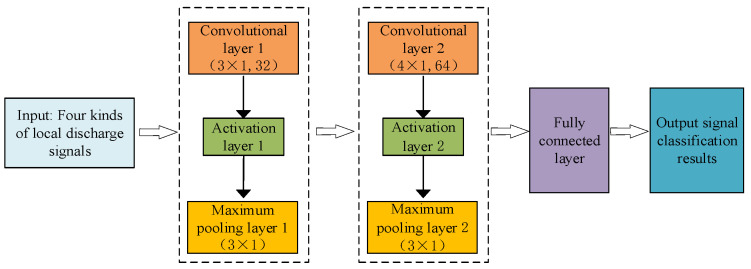
CNN-based cable terminal discharge classification model of high-speed EMU.

The CNN primarily consists of three fundamental components: convolutional layers, pooling layers, and fully connected layers [51]. In this study, a CNN-based classification model is employed to categorize the discharge signals from four typical defects in high-speed EMU cables. This model is constructed using two convolutional layers, two activation layers, two maximum pooling layers, and one fully connected layer; its block diagram is illustrated in Figure 5.

In this study, the discharge signal from the terminal of the high-speed EMU cable serves as the input to the model. The convolutional and pooling layers extract features and compress this information into a feature map form. For the first convolutional layer, the kernel size is set to 3 × 1 with 32 kernels, and the step size is 1. For the second convolutional layer, the kernel size is adjusted to 4 × 1 with 64 kernels, maintaining the step size at 1. The first maximum pooling layer has a kernel size of 3 × 1 and a step size of 1, mirroring the settings of the second maximum pooling layer, which also adopts a kernel size of 3 × 1 and a step size of 1.

Convolution Layer: This layer applies the convolution operation across local regions of the input using the convolution kernel of the filter, aiming to extract local features effectively. The convolution layer employs weight sharing, significantly reducing the number of learning parameters and thereby mitigating the risk of overfitting [52]. Each convolutional layer in the network comprises multiple convolutional kernels, with the parameters of each kernel being optimized through the backpropagation algorithm [53]. Each convolution can be performed on the input sequence by convolving the equation as follows:

(1) $$y_{m}^{k}(t)=w^{\left(k\right)}*x_{m}=\sum_{i=1}^{L}w^{\left(k\right)}(i)x_{m}(t-i+1)$$

where *x_m_* is the input sequence, *w*^(*k*)^ is the weight of the *k* th convolution kernel, and the size is L.

Activation Layer: The output from the convolutional layer serves as the input to the activation function. The role of the activation function is to transform the output of the convolutional layer in a nonlinear manner. This transformation enhances the linear separability of data that was originally scattered. Commonly used activation functions in CNNs include the sigmoid function, the tanh function, and the rectified linear unit (ReLU) function [54]. The expressions for these three functions are given as (2)–(4), respectively. Compared with the other two activation functions, the ReLU function can effectively reduce the amount of computation and improve the expression ability of the network by debugging the activity of neurons in the network [54]. Therefore, the ReLU function is used as the activation function of the activation layer in this study.

(2) $$y=\frac{1}{1+e^{-x}}$$

(3) $$y=\frac{e^{x}-e^{-x}}{e^{x}+e^{-x}}$$

(4) y=max{0,x}

where *x* is the output data of the convolution layer.

Pooling Layer: Following the convolution operation, the quantity of extracted feature sequences increases, leading to an expansion in data dimensions and a rise in computational complexity. The pooling layer serves to reduce the data width and the number of network parameters, thus lowering computational costs and helping to prevent overfitting [55]. There are two common pooling functions: average pooling and max pooling. The average pooling computes the mean of the input data to serve as the output of the layer, while the max pooling selects the maximum value from the input data as the output [56]. The expressions of average pooling and max pooling are given as (5) and (6), respectively. The max pooling is particularly effective in capturing important local features of the data, thereby improving the recognition accuracy of the model. Consequently, the max pooling function is adopted in this study.

(5) $$y_{c}^{h+1}(i)=\frac{1}{w}\sum_{(i-1)w}^{iw}x_{c}^{h}(t)$$

(6) $$y_{c}^{h+1}(i)=\underset{(i-1)p\leq t\leq ip}{\max}x_{c}^{h}(t)$$

where *w* is the width of pooling layer, $x_{c}^{h}(t)$ is the value of the *t*th neuron in the c eigenvalue of the *h*th layer, and $y_{c}^{h+1}$ is the value of neurons in layer *h* + 1.

Fully Connected Layer: The role of this layer is to integrate and refine the features extracted by the alternating convolution and pooling layers. This is achieved by flattening the output feature map from the final convolution or pooling layer into a one-dimensional feature vector, which allows for further feature extraction [57]. Then, the fully connected layer maps these extracted feature vectors to the sample label space and classifies them by constructing a classifier. For the classification, the softmax function is usually selected as the activation function of the fully connected layer, which converts the output vector into a set of probability distributions, according to which the model makes category prediction and selects the category with the highest probability as the output [58].

(7) $$P_{j}=\mathrm{softmax}(r_{i})=\frac{e^{r_{j}}}{\sum_{i=1}^{k}e^{r_{i}}}$$

where *P_j_* is the probability of belonging to the correct class, *r_j_* is the node value of the *j*th neuron, and *k* is the total number of classes.

### 3.2. PD Data for Training and Verification

**Figure 6:**
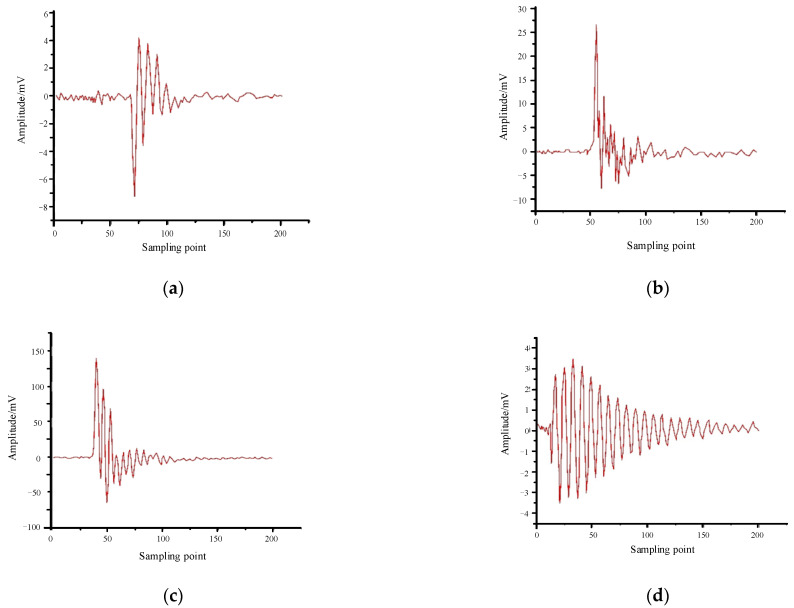
Time-domain diagram of discharge high-frequency pulse current signals of four defect models: (**a**) tip discharge, (**b**) surface discharge, (**c**) air gap discharge, and (**d**) suspension discharge.

Through the PD test platform shown in Figure 1, the PD signals from high-speed EMUs with various defect models are collected; from these collected PD signals, single-wave time-domain signals for four types of PD signals associated with the defect models are extracted, as illustrated in Figure 6.

To ensure the richness and representativeness of the data, 400 sets of four kinds of PD signals are extracted from the collected signals. Out of the 1600 total sets of data, 1200 sets are randomly selected to train the model. The remaining 400 sets are used to verify the model’s accuracy in identifying different types of defects.

### 3.3. Classification Steps

**Figure 7:**
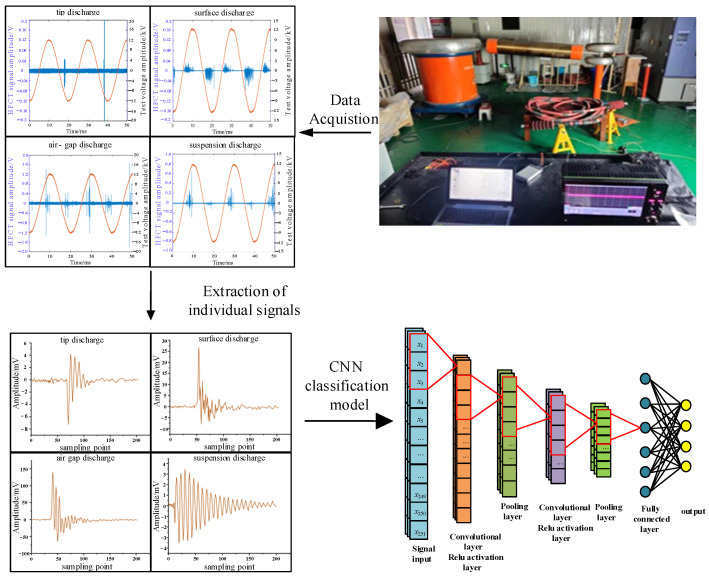
Classification steps based on the CNN.

The classification process of the proposed CNN-based model is illustrated in Figure 7 and can be summarized in the following steps:

1. Signal acquisition: A test platform is built, and cable terminal models with four types of defects are created. The HFCT is used to measure the PD signals of the cable terminals.
2. Dataset construction: Four different types of discharge signals are collected, and a single signal is extracted. For each of the four signals, 251 sampling points are selected, resulting in 400 sets of data for each type. Out of these 1600 datasets, 1200 are randomly chosen to construct the training dataset, while the remaining 400 sets are designated as the test dataset.
3. Data normalization: To simplify the data complexity, disparate data in the set are processed. This step facilitates faster gradient descent, aiding in finding the optimal solution and enhancing the model’s accuracy and convergence speed. The dataset from step 2 is normalized using the following expression.
  (8) $$Y_{i}=\frac{y_{i}-y_{\min}}{y_{\max}-y_{\min}}$$
  where *y_i_* is the sample value before normalization, *Y_i_* is the normalized sample value. *y*_min_ is the minimum value of the sample, and *y*_max_ is the maximum value of the sample.
4. CNN training and classification: The normalized dataset from step 3 serves as the input to the constructed CNN-based model. After processing through two convolutional layers, two pooling layers, and one fully connected layer, the classification results are produced.

## 4. Results Analysis

The models discussed in this paper are implemented using the MATLAB 2021a software, installed on a personal computer equipped with an Intel i5-10210U CPU (1.6 GHz clock frequency) and 8 GB of RAM.

In this study, training loss and accuracy are used to evaluate the recognition effectiveness of CNN. Loss quantifies the discrepancy between the predicted value and the true value. Cross entropy is used as the loss function to describe the gap between the probability distribution of the predicted values and the actual values. This measure reflects the model’s degree of fit [59]. The expression for cross entropy is written as

(9) $$Loss=-\frac{1}{N}\sum_{n=1}^{N}\sum_{i=1}^{k}y_{i}^{\left(n\right)}\log(p_{i}^{\left(n\right)})$$

where *y_i_* is the label value of sample *i*, *p_i_* is the probability of correctly predicting the sample, and *N* is the total number of samples.

Four performance indices, namely accuracy, precision, recall, and F1-score, are utilized to evaluate the effectiveness of classification. Taking binary classification as an example, accuracy measures the proportion of correctly predicted classes, precision assesses the percentage of actual positives among all samples predicted as positive, recall calculates the proportion of actual positive samples correctly identified relative to all actual positives, and F1-score reflects the balance between precision and recall. The formulas for these indices are provided in (10)–(13), and Table 2 illustrates the meanings of each term used in the formulas. Regarding the learning rate reduction strategy, the adaptive moment estimation (Adam) optimizer [59] is employed to decrease the learning rate to one-tenth of its value every 10 training iterations.

(10) $$\mathrm{Accuracy}=\frac{T_{N}+T_{P}}{T_{N}+F_{N}+T_{P}+F_{P}}$$

(11) $$\mathrm{Precision}=\frac{T_{P}}{T_{P}+F_{P}}$$

(12) $$\mathrm{Recall}=\frac{T_{P}}{F_{N}+T_{P}}$$

(13) $$F1\mathrm{-score}=\frac{2\times \mathrm{Precision}\times \mathrm{Recall}}{\mathrm{Precision}+\mathrm{Recall}}$$

### 4.1. Influence of Different Optimizers

By calculating network parameters that influence model training and output, optimizers aim to minimize the loss function, guiding it toward an optimal value [42]. This study discusses the effects of the Adam optimizer, the stochastic gradient descent with momentum (SGDM), and the root mean square propagation (RMSprop) optimizers on the CNN-based classification model to identify the most suitable optimizer. In total, 75% of the data is randomly selected from the PD dataset to construct the training dataset, with the remaining data serving as the test dataset.

**Figure 8:**
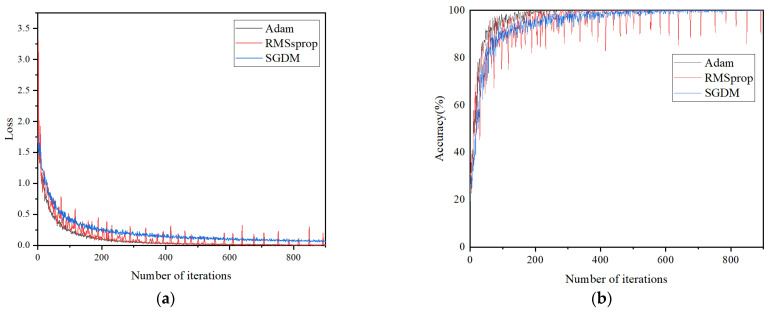
Training accuracy and loss of three optimizers: (**a**) loss curve and (**b**) accuracy curve.

**Figure 9:**
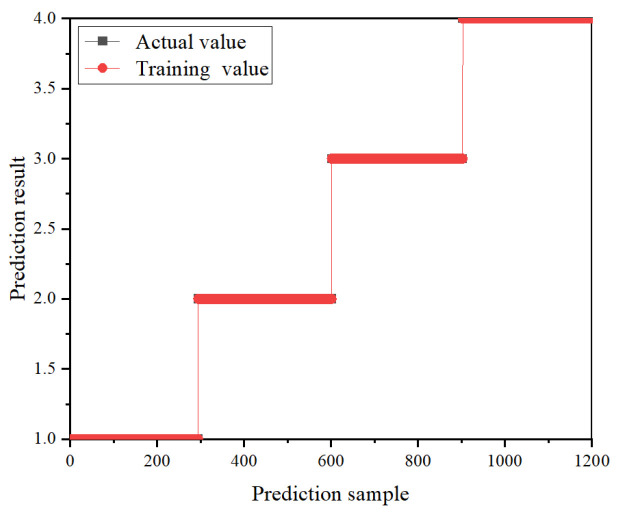
Training results based on three optimizers.

**Figure 10:**
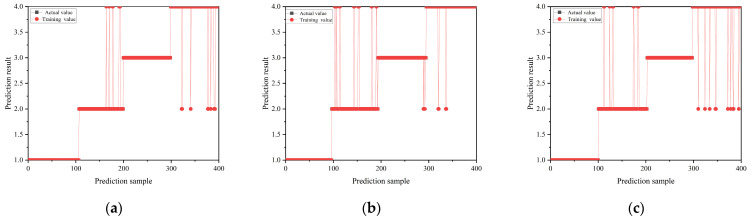
Classification of terminal PD of high-speed EMU cables based on three optimizers: (**a**) Adam, (**b**) RMSprop, and (**c**) SGDM.

The evaluation focuses on the impacts of three optimizers on the model performance and accuracy. The training accuracy and loss curves of the CNN-based classification model, utilizing these three optimizers through the iterative process, are depicted in Figure 8. In the training process, the recognition accuracy and classification accuracy of the model based on three different optimizers for the four discharge signals are shown in Figure 9 and Figure 10, showing the classification results of the three optimizers for the four signals in the test set. Table 3 shows the classification accuracy of the CNN-based classification model, using the three different optimizers, for the four discharge signals.

As shown in Figure 8, the CNN-based classification model employing the Adam optimizer converges fastest and exhibits a smooth curve, indicating efficient learning. In contrast, the model using the SGDM optimizer shows the slowest convergence, whereas the RMSprop optimizer achieves better convergence than SGDM but with more considerable curve fluctuation, indicating less stability in the trained model. According to Figure 9, all three optimizer-based models achieve 100% signal classification accuracy during training. Combining the results from Figure 10 and Table 3, the average accuracy of the CNN-based classification model, based on the three optimizers for identifying the four defects, ranks as follows: Adam > SGDM > RMSprop. This suggests that the CNN-based classification model using the Adam optimizer demonstrates superior learning performance and classification effectiveness. Consequently, the Adam optimizer is selected as the optimizer for the cable terminal discharge classification model in high-speed EMUs for this study.

### 4.2. Influence of Different Training Data Amounts

**Figure 11:**
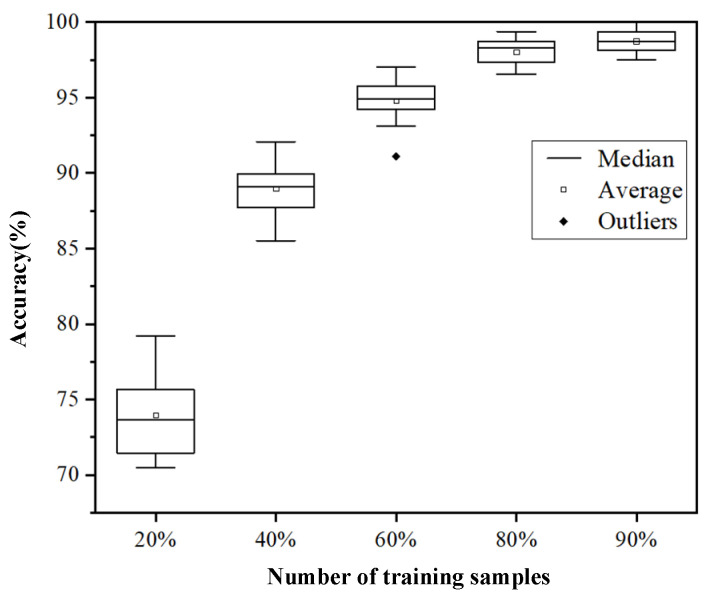
Box plot of classification accuracy based on different training data amounts.

**Figure 12:**
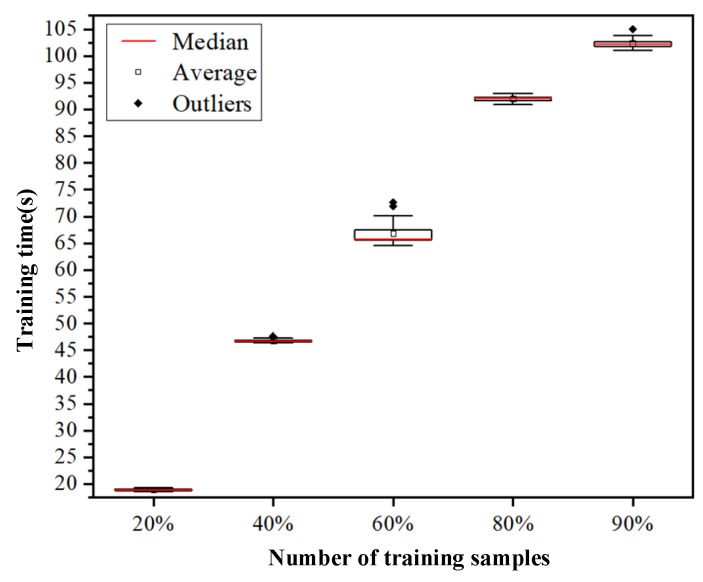
Box plot of training time based on different training data amounts.

The first 20%, 40%, 60%, 80%, and 90% of the PD data of high-speed EMU cable terminals are used as training datasets, and the rest are used as validation datasets. The CNN-based classification model proposed in this paper was used to test the five datasets, and the influence of different amounts of training data on the accuracy of the model was studied. Each case was verified 100 times, and the classification accuracy and training time of 100 times were obtained. Figure 11 shows the box diagram drawn according to the classification accuracy of the model under 100 tests, and Figure 12 shows the box diagram drawn according to the training time of the model. Table 4 shows the average precision of the model’s recognition of each discharge signal under 100 tests, and the average accuracy of the model under 100 tests, which corresponds to the mean value of the box chart in Figure 11.

Table 4 reveals that the classification accuracy for the four types of discharge in the cable terminal discharge signal classification model of high-speed EMUs improves with an increase in training data volume. Furthermore, Figure 11 demonstrates that as the training data volume expands, the range of recognition accuracy for the CNN-based classification model narrows, and the average accuracy rate approaches nearly 100%. More training data introduce greater data diversity, enhancing the model’s robustness and minimizing performance variances across different data subsets. This leads to a more concentrated range of high accuracy.

Moreover, as can be seen from Figure 12, the model’s training time also escalates with larger training data volumes. The average training time for 100 sessions across the five data groups was recorded as 18.91 s, 46.78 s, 66.84 s, 92.03 s, and 102.38 s, respectively.

More training data require more forward-propagation and back-propagation calculations so that the model can better understand and fit the distribution of the data, which helps to improve the algorithm, improve the generalization ability and performance of the model, and will also require more running time.

### 4.3. Comparison of CNN with Other Classification Models

The proposed CNN-based classification model is compared with the BPNN-based and RBFNN-based classification models. In total, 75% of the PD dataset is utilized to train these three models, and the remaining data are employed to assess the models’ recognition capabilities. For the BPNN, the number of nodes in the input layer is set to 251, the number of nodes in the hidden layer is set to 6, and the number of nodes in the output layer is set to 4. In addition, the target error of the model is set to 1 × 10^−6^, and the learning rate is 0.01. For the RBFNN, the Gaussian kernel is used. The RBF can be adaptively determined according to the training data, and the expansion speed of the RBF is set to 100.

**Figure 13:**
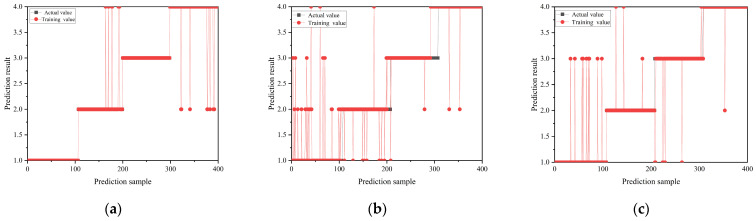
Identification results of the three NN-based classification models: (**a**) CNN, (**b**) RBFNN, and (**c**) BPNN.

The comparison results are depicted in Figure 13, while Table 5 and Table 6 show the accuracy, precision, recall rate, and F1-score of the three models under different types of discharge signals. It can be seen from Table 5 and Table 6 that the CNN model has higher recognition precision, recall rate, and F1-score for the four discharge signals. Although the recognition effect of the discharge at the tip and the discharge at the air gap is relatively poor, the accuracy rate of the CNN model is higher than that of the other two models, indicating that the model has a more accurate overall prediction of the four discharge signals, better classification effect, and more stable model.

Although the proposed CNN-based classification model demonstrates higher accuracy than the other two NN-based classification models, it also requires a longer runtime. This is attributed to CNN’s convolution layer, which enhances the model’s local feature extraction capability by using convolution kernels to capture specific signal features, thereby effectively differentiating between different signal types. The increased sensitivity and accuracy of signal recognition comes at the cost of increased computational complexity, resulting in higher time costs.

## 5. Conclusions

In this paper, a CNN-based classification method for distinguishing different defect discharge signals in high-speed EMU cable terminals is presented. Within a laboratory environment, a PD test platform using the HFCT was established for the collection of PD signals from various defects, and these signals were classified using the proposed CNN-based classification model. Furthermore, the effects of three different optimizers and varying amounts of training data on the classification accuracy of the high-speed EMU cable terminal PD model were investigated, and the proposed CNN-based classification model was compared with two existing NN-based classification models. The main conclusions are drawn as follows:

1. Compared with SGDM and RMSprop optimizers, the Adam optimizer shows lower loss and higher classification accuracy in CNN-based classification model training, and the training effect is more stable.
2. It is found that increasing the amount of training data can enhance the robustness of the model and improve the classification accuracy but at the cost of increasing the training time.
3. Compared with the BPNN-based and RBFNN-based classification models, the CNN-based classification model proposed in this paper shows higher classification accuracy and can identify four different types of defects more accurately.

The method proposed in this paper can avoid the process of learning manual feature extraction in traditional machine learning and can effectively identify the discharge signals of four different defect types and achieve a high classification accuracy. In future research, we will focus more on artificial intelligence technology to optimize and perfect our classification model by learning and exploring new methods so that we can build a model with better classification effect and more stable performance.

## Figures and Tables

**Table 1:** Advantages and disadvantages of PD detection methods and their application scopes.

Detection Method	Major Advantage	Major Defect	Main Applicable Scopes
Pulse current method	High sensitivity	Limited anti-interference capability	Offline measurement
Chemical detection method	Strong anti-interference	Challenges in online gas component extraction	Oil filling equipment
Radio frequency detection method	High sensitivity without affecting equipment operation	Limited anti-interference capability	Online measurement
Infrared imaging	High sensitivity	Incomplete detection	Electrical equipment
Flash spotting	Strong resistance to electromagnetic interference	Expensive	Laboratory research
High-frequency pulse current method	High sensitivity; easy to install	Susceptible to ground commutation	High voltage cables and electrical equipment
Ultrasonic method	Strong resistance to electromagnetic interference	Average sensitivity	Electrical primary equipment
Ultra-high frequency method	High sensitivity	Limitations in quantifying	Electrical equipment such as transformers

**Table 2:** Confusion matrix.

	Predicted	0	1
Actual			
0		$T_{N}$	$F_{P}$
1		$F_{N}$	$T_{P}$

**Table 3:** Classification accuracy of cable terminal discharge by different optimizers.

Discharge Type	Adam		RMSprop		SGDM	
	Precision (%)	Accuracy (%)	Precision (%)	Accuracy (%)	Precision (%)	Accuracy (%)
Surface Discharge	100	95.8	100	94.5	100	95
Tip Discharge	92.5		84.4		90.1	
Suspended Discharge	100		98.1		100	
Air Gap Discharge	91.2		96.3		90.1	

**Table 4:** Classification results of terminal discharge of high-speed EMU cables based on different training data.

Training Data Volume	Surface Discharge	Tip Discharge	Suspended Discharge	Air Gap Discharge	Accuracy
20%	74.2%	63.4%	85.6%	71.4%	73.6%
40%	95.4%	79.9%	96.7%	81.8%	88.5%
60%	99.2%	90.2%	99.6%	89.8%	94.7%
80%	100%	95.8%	100%	93.8%	97.4%
90%	100%	97.9%	100%	96.8%	98.7%

**Table 5:** Classification results of terminal discharge of high-speed EMU cables with different models: accuracy and precision.

Discharge Type	CNN		RBFNN		BPNN	
	Precision (%)	Accuracy (%)	Precision (%)	Accuracy (%)	Precision (%)	Accuracy (%)
Surface Discharge	100	95.8	80.2	91.2	91.6	94.8
Tip Discharge	92.5		89.7		97	
Suspended Discharge	100		96.8		93	
Air Gap Discharge	91.2		98.2		97.8	

**Table 6:** Classification results of terminal discharge of high-speed EMU cables with different models: recall and F1-score.

Discharge Type	CNN		RBFNN		BPNN	
	Recall (%)	F1-Score (%)	Recall (%)	F1-Score (%)	Recall (%)	F1-Score (%)
Surface Discharge	100	100	100	89	93.3	92.4
Tip Discharge	90.8	91.6	85	87.3	97	97
Suspended Discharge	100	100	95.6	96.2	91.1	92
Air Gap Discharge	92.7	92	86.7	92.1	97.3	97.5

## Data Availability

The data presented in this study are available upon request from the corresponding authors. The data are not publicly available due to further ongoing analysis, but data sharing may be anticipated upon completion.
